# Determinants of household catastrophic costs for drug sensitive tuberculosis patients in Kenya

**DOI:** 10.1186/s40249-021-00879-4

**Published:** 2021-07-05

**Authors:** Beatrice Kirubi, Jane Ong’ang’o, Peter Nguhiu, Knut Lönnroth, Aiban Rono, Kristi Sidney-Annerstedt

**Affiliations:** 1 Department of Global Public Health, Karolinska Institutet, Stockholm, Sweden; 2 WHO Collaborating Centre for Tuberculosis and Social Medicine, Stockholm, Sweden; 3 The Health and Social Protection Action Research & Knowledge Sharing Network (SPARKS), Stockholm, Sweden; 4 Centre for Respiratory Disease Research, Kenya Medical Research Institute, Nairobi, Kenya; 5 Health Economics Research Unit, KEMRI-Wellcome Trust Research Program, Nairobi, Kenya; 6 Monitoring, Evaluation & Research, National Tuberculosis, Leprosy and Lung Disease Program, Ministry of Health, Nairobi, Kenya

**Keywords:** Tuberculosis, Health expenditure, Income loss, Social protection, Kenya

## Abstract

**Background:**

Despite free diagnosis and treatment for tuberculosis (TB), the costs during treatment impose a significant financial burden on patients and their households. The study sought to identify the determinants for catastrophic costs among patients with drug-sensitive TB (DSTB) and their households in Kenya.

**Methods:**

The data was collected during the 2017 Kenya national patient cost survey from a nationally representative sample (*n* = 1071). Treatment related costs and productivity losses were estimated. Total costs exceeding 20% of household income were defined as catastrophic and used as the outcome. Multivariable Poisson regression analysis was performed to measure the association between selected individual, household and disease characteristics and occurrence of catastrophic costs. A deterministic sensitivity analysis was carried using different thresholds and the significant predictors were explored.

**Results:**

The proportion of catastrophic costs among DSTB patients was 27% (*n* = 294). Patients with catastrophic costs had higher median productivity losses, 39 h [interquartile range (IQR): 20–104], and total median costs of USD 567 (IQR: 299–1144). The incidence of catastrophic costs had a dose response with household expenditure. The poorest quintile was 6.2 times [95% confidence intervals (*CI*): 4.0–9.7] more likely to incur catastrophic costs compared to the richest. The prevalence of catastrophic costs decreased with increasing household expenditure quintiles (proportion of catastrophic costs: 59.7%, 32.9%, 23.6%, 15.9%, and 9.5%) from the lowest quintile (Q1) to the highest quintile (Q5). Other determinants included hospitalization: prevalence ratio (PR) = 2.8 (95% *CI:* 1.8–4.5) and delayed treatment: PR = 1.5 (95% *CI:* 1.3–1.7). Protective factors included receiving care at a public health facility: PR = 0.8 (95% *CI:* 0.6–1.0), and a higher body mass index (BMI): PR = 0.97 (95% *CI:* 0.96–0.98). Pre TB expenditure, hospitalization and BMI were significant predictors in all sensitivity analysis scenarios.

**Conclusions:**

There are significant inequities in the occurrence of catastrophic costs. Social protection interventions in addition to existing medical and public health interventions are important to implement for patients most at risk of incurring catastrophic costs.

**Graphic abstract:**

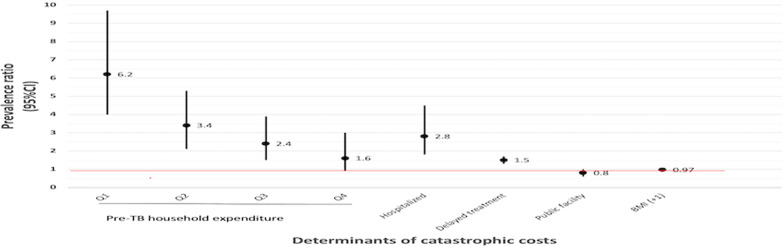

**Supplementary Information:**

The online version contains supplementary material available at 10.1186/s40249-021-00879-4.

## Background

Tuberculosis (TB) is a public health concern globally and a major cause of morbidity and mortality [1]. Globally, TB is the leading cause of death from a single infectious agent [1]. TB and poverty form a vicious cycle. TB disproportionately affects the poor [2]. Poverty increases the risk of TB infection associated with overcrowding, malnutrition and poor sanitation [3, 4]. On the other hand, poor patients face barriers in accessing care, resulting in more severe disease and inability to work [2, 5]. As a result, households may engage in coping mechanisms such as dissaving, i.e. selling assets or seeking loans to support their treatment, causing further impoverishment [6–8] perpetuating the medical poverty trap [9].

The World Health Organization (WHO) End TB Strategy aims to end the global TB epidemic [10]. The strategy identifies one of the key targets as reducing the number of TB-affected households facing catastrophic costs to zero [10]. WHO developed a TB Patient Cost Survey (PCS) manual/workbook that guides assessment of costs relative to household income, and estimates the proportion of patients experiencing catastrophic costs, exceeding a threshold of 20% of household income [10, 11]. Catastrophic costs are also associated with adverse TB outcomes such as loss to follow up, low uptake of preventive treatment for contacts, low HIV testing rates or even death [12].

In order to address equitable financial protection for TB patients, there is need to extend coverage of mitigative interventions for patients with TB. The poor and patients with drug resistant TB are more vulnerable to higher costs due to TB [7, 12, 13]. Patients with drug resistant TB (DRTB) tend to experience higher treatment costs associated with more severe disease manifestation, prolonged treatment and treatment strategies and are therefore in need of special support to reduce the risk of catastrophic costs [7, 13, 14]. While drug-sensitive TB (DSTB) disease is less fatal and has considerably less costs compared to DRTB patients, the sheer number of patients is high, with significant societal implications of catastrophic costs [1]. Consequences of incomplete DSTB treatment include failed treatment, developing drug resistance, heavy economic and social burden on affected households and even death [15, 16].

Kenya is one of the top 20 countries contributing to the highest burden of TB, TB/HIV co-infection and DRTB globally [1]. The Kenya national TB prevalence survey conducted in 2015, estimated a TB prevalence of 558 cases per 100 000 population [17]. The disease disproportionately affects productive age groups, males and those residing in the urban areas [1, 17]. A summary of the country disease burden is described in Additional file 1.

TB services are provided at all levels of the healthcare system. Fifty-eight percent of DSTB patients initiated care at primary healthcare facilities [18]. The referral pathways ensure that sicker patients are treated in higher level facilities [19]. The TB treatment model is predominantly community DOTS with planned visits to the facility [15]. In the intensive phase, the patient has two weekly visits, which is less frequent in the continuation phase. This may differ for patients whose condition deteriorates, develop severe side effects or have other comorbidities. Patients who require critical care are hospitalized for inpatient care until they are stable to continue treatment at home.

The National TB Programme (NTP) provides TB diagnosis through sputum examination and anti-TB medicines at no cost to the patient [19]. This excludes radiological examinations (X-Rays) [19]. The private sector also plays a key role in TB service delivery [18]. Between 2013 and 2017, 20% of TB patients received care in private hospitals [20]. The private sector includes for profit hospitals, faith-based organizations (FBOs), and non-governmental organizations (NGOs). The NTP supports patients in private facilities through subsidized diagnosis and free medicines, as in public hospitals [19]. However, the cost of other complementary tests and treatment is borne by the patient.

In 2016, Kenya conducted the first national TB PCS to establish the costs incurred by TB patients and their households, from the onset of TB illness to treatment completion [21]. The survey estimated that 27% of all TB patients incurred catastrophic costs [21]. When considering only those with drug-resistant TB, 86% incurred catastrophic costs.

Social support interventions for TB patients in Kenya exclude those with DSTB. The NTP prioritizes DRTB patients for support through cash transfers and health insurance [22]. This is limited; corresponding to 470 DRTB patients out of 95 000 TB cases notified in Kenya in 2018 [1]. Following the Kenya PCS, consultations on the need to extend this coverage led to the development of the TB social protection policy targeting patients with DSTB [23].

In the context of limited resources, there is need to prioritize DSTB patients and households who are most vulnerable to catastrophic costs for targeted interventions. However, given resource constraints, there may be a need for special targeting patients who have DSTB. There is a gap in identifying predictors for catastrophic costs among patients on treatment for DSTB. While, several PCSs have reported the determinants of catastrophic costs for different countries [21, 24], they often combine all patients [21] or only consider costs incurred in the pre-diagnosis period [24]. Identifying the predictors for catastrophic costs among DSTB patients, would be the first step in detecting vulnerable households. Hence the aim of this study was to identify the determinants for catastrophic costs among patients with drug-sensitive TB (DSTB) and their households in Kenya.

## Methods

### Study setting

Kenya is a lower, middle-income country with a population of more than 47 million, of whom 69% reside in rural areas [25]. The country’s gross domestic product (GDP) per capita is US Dollar (USD) 1710 [26]. With a Gini coefficient of 0.4, about 36% of the population lives below the international poverty line, spending less than USD 1.9 per day (19). In 2018, the human development index (HDI) was estimated at 0.58 placing it in the medium human development category [27]. The country is administratively divided into 47 counties, with varying population density, and further into sub-counties for service provision [25, 28]. The health care delivery system including the TB service provision follows the administrative structure with referral mechanisms in place [28].

The first Kenya national patient cost survey was conducted in 2016 [21]. The sample was nationally representative and included patients from 111 pre-selected health facilities of 30 counties [21]. The data was collected between May and June 2017, using a structured questionnaire adapted from the WHO costing tool [11]. A more detailed description of the survey recruitment, methods and results is available elsewhere [21]. This national survey provides the data for our study.

### Study design and population

The study design involved a secondary analysis of quantitative data. The data included all DSTB patients (adults and children) collected during the 2016 Kenya national TB PCS in the dataset [21]. The study excluded patients who had not completed two weeks in the reported phase of treatment and those on treatment for DRTB. Prior to data extraction, we developed a list of possible variables to enable us to answer the research question based on literature. We used the Kenya PCS questionnaire and selected the list of variables found in the database. The available data was based on the TB category group and the phase of treatment (see Additional file 2).

### Cost measures

The study outcome variable was the binary presence of catastrophic costs. To determine catastrophic costs, the proportion of annual household expenditure spent on total TB related costs (direct and indirect costs) was calculated. The cost was considered catastrophic when this proportion exceeded the threshold of 20% [10]. Operational definitions of key concepts of TB disease, treatment and costs as used in this study are described in the glossary (Box 1).

**Box 1:** Glossary of operational definitions for tuberculosis disease, treatment and costs

*Patient registration group:* Classification of TB based on history of previous TB treatment
New case: Patient who has never been treated for TB before
Retreatment case: Patient treated with anti-TB drugs in the past and are now on treatment after the following
**∙** Relapse (previously completed treatment/cured)
**∙** Treatment failure (last treatment failed at the end of treatment)
**∙** Loss to follow up (patients who did not complete their last treatment)
*TB treatment phases*:
Intensive phase: the first two consecutive months of TB treatment
Continuation phase: the four consecutive months immediately following the intensive treatment phase
During treatment: the period of time spanning from the beginning of the intensive treatment phase to the end of continuation treatment phase
*Model of care*:
Community Directly Observed Treatment Strategy (DOTS): Patient receives ambulatory care; collects medicine from the health facility biweekly or weekly
Facility based DOTS: Patient receives medicine at the health facility under observation, usually on a daily basis
Hospitalization: Patient is treated as an inpatient until symptoms are stabilized
*DOTS supporter/treatment supporter*:
Refers to person, often family member who accompanies the patient to the hospital or stays with the patient during a hospitalization event. Could also be the DOTS supporter i.e. observes/reminds patient to take medicine at the specified time
*Treatment delay*:
Time between the onset of symptoms and the start of treatment. For this study the threshold is 4 weeks
*TB costs*
**∙** Direct medical costs: costs of medical examinations and medicines, including hospitalization
**∙ **Direct non-medical expenses: costs of, TB-care-related transport, accommodation, natural remedies and other miscellaneous expenses. Also includes costs incurred by guardian or treatment supporter
**∙ **Direct costs: the sum of the direct medical costs and direct non-medical costs (above)
**∙ **Lost income (indirect costs): the income the patient estimated that the household lost due to TB illness or when seeking care/treatment. This also includes costs incurred from onset of symptoms and prior to presentation for TB diagnosis
**∙ **Total costs: direct costs plus lost income
*Household (HH)*:
People that live together under the same roof, often with family relations, and share at least one meal a day

A patient’s perspective was assumed in the estimation of total costs. Direct costs were a total of patient medical costs, patient non-medical costs and guardian and/or treatment supporter costs. Medical costs included consultation fees, payment for laboratory and imaging tests, hospitalization costs and any out-of-pocket (OOP) costs incurred towards purchasing drugs. Non-medical direct costs included OOP payments for travel costs, accommodation costs, food supplements, traditional medicine and costs for services provided by traditional healers. Guardian and treatment supporter costs included OOP payments for transport, accommodation and valuation of time lost. Time lost was valued using the human capital approach by multiplying total hours lost by patient due to the illness or for TB care by the hourly wage calculated from the reported income. The 2017 Kenyan government stipulated minimum wage was adopted when no income was reported. The patient’s valuation of productivity loss was considered as indirect costs [11].

A two-step procedure was used to calculate the costs. First, the two-week estimates reported from the survey were scaled up to estimate the costs and time loss for the treatment phase at the time of the interview. Since patient costs were only available for one phase of treatment, cost for missing phase were then estimated using cost data from the patients in the alternative phase. Costs were extrapolated based on the median costs incurred by the patients in the treatment phase in question at the time of the interview. Hence, the cost and time lost information for those in intensive phase, were used to project intensive phase costs for those interviewed in the continuation phase and vice versa for those in the intensive phase. The mean imputation approach was used to handle missing data [29].

To estimate the annual household expenditure, a consumption approach was used based on self-reported amounts spent on selected goods and services such as food items, non-food items, durable goods and housing [11]. This approach was selected as many participants do not have a formal income. The total household expenditure was converted to the USD 2020 value [30, 31].

Recognizing the inadequacies of data collected during a cross-sectional survey, several assumptions were made in order to determine the costs incurred, productivity losses and household expenditure (Box 2).

**Box 2:** List of assumptions for the estimation of costs

*Regarding the patient condition*:
1. That all patients remained in the same treatment category (DSTB) until the end of their current treatment
2. That all patients adhered to the NTP recommended visit schedule for the duration of treatment (biweekly in intensive phase and once a month in the continuation phase)
3. No further hospitalization episodes happened after the interview
*Regarding the estimation of costs*:
4. The costs incurred by patients interviewed in intensive phase were similar to those incurred previously by those interviewed in continuation phase given similar characteristics: demographic, disease and treatment categories, and socioeconomic characteristics. These characteristics were used to estimate costs and productivity losses for the alternative (missing) phase of treatment
5. Likewise, costs incurred by patients interviewed in continuation phase were similar to those to be incurred in future by those interviewed in the Intensive phase given some similar characteristics as above
6. That the costs and productivity losses remained constant for the entire treatment phase
7. Where the patient was a child, the costs and productivity losses considered were those of the parent or primary guardian who was also assumed to be the treatment supporter
8. To value the time lost by guardians and treatment supporters using human capital approach, it was assumed that all workers earned the 2017 Kenya government stipulated minimum wage; which is standardized by urban or rural residence
9. Household expenditure remained constant despite income changes due to TB [32]
10. Discounting was not taken into consideration since the time horizon is 1 year

*DSTB* drug-sensitive tuberculosis, *NTP* National TB Programme, *TB* tuberculosis

### Data analysis

**Fig. 1:**
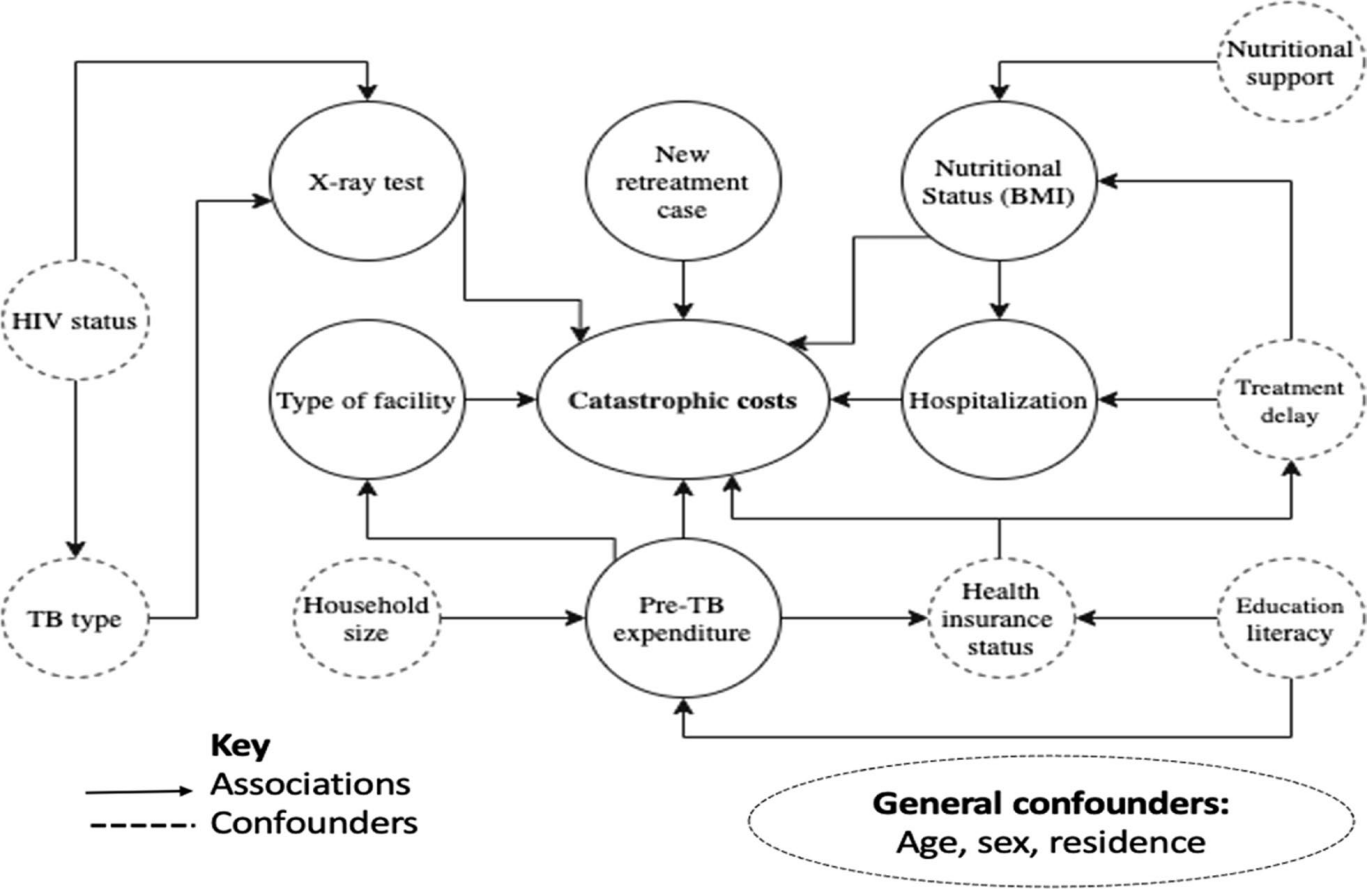
Conceptual framework of selected exposure variables and proposed associations based on the literature

The exposure variables were selected based on existing literature. Several complex relationships of covariates exist based on previous literature as depicted in Fig. 1.

The exposure variables were grouped into three categories: individual, household, and disease and care-seeking characteristics [21, 33–35]. Individual factors describe the patient characteristics, household factors describe characteristics that are influenced by their next of kin or who they live with and TB related characteristics are related to the disease type, severity and treatment. The exposure variables are described in further detail in Table 1.

**Table 1:** A summary of exposure variables and their measures

Type of data	Category	Variable	Definition	Measure
Continuous	Individual characteristics	Age	Age of patient in years rounded off to the next whole unit	Years
		BMI	Nutritional status of patient	Body weight (kilograms)/height^2^ (metres)
	Household characteristics	Household size	Total number of people living together and sharing at least one meal/day (usually dinner)	Units
		Pre-TB expenditure	Total annual household expenditure (patient and/or others) prior to falling ill with TB	
Categorical	Individual characteristics	Gender	Gender of patient	Male/Female
		Diabetes	Type II diabetes mellitus as a comorbidity	Yes/No
		HIV status	Presence of HIV coinfection	Yes/No/Undisclosed
		Education	Patient’s level of education	No education, primary education or lower (< 8 years), secondary education and above =/> 8 years in school
		Nutritional Support	Receiving nutritional support	Yes/No
	Household characteristics	Residence	Living in urban/rural area?	Urban/Rural
		Insurance status	Does the patient have health insurance coverage?	No/Social health insurance/Private health Insurance
	Disease & care-seeking characteristics	X-ray test	Radiography test done for diagnosis of TB	Yes/No
		Hospitalized	Any hospitalization during current TB treatment	Yes/No
		TB type	Disease affecting the chest or another body organ?	Pulmonary/Extrapulmonary
		Facility type	Type of health facility giving care	Public/Private
		Retreatment	First episode of treatment or not?	New/Retreatment & Relapse
		Treatment delay	Length of the delay between onset of symptoms and start of treatment	< 4 weeks/4 weeks or more delay

*BMI* body mass index, *TB* tuberculosis

The variables age and BMI were treated as continuous variables for the analysis. Pre-TB household expenditure variable was divided into quintiles in order to demonstrate the dose response relationship between the economic status and catastrophic costs. The mean imputation approach was used for missing variables related to the patient costs [29].

To define the participant characteristics, descriptive statistics [i.e. mean, standard deviation (SD), median, interquartile ranges (IQR) and proportions] were used. A comparison of patients who incurred catastrophic costs and those without catastrophic costs was conducted using Pearson’s chi square, Fishers test, Student’s *t* test and Mann–Whitney test as appropriate. Statistical significance was defined as *P* < 0.05. The analysis took into account sampling weights used for the Kenya PCS to adjust for clustering effects related to the sampling method [21]. Data analysis was done using Stata statistical software 15 (College Station, TX: StataCorp LLC) [36].

Univariable regression analysis was used describe the association between the individual exposure variables and the outcome variable (occurrence of catastrophic costs). We investigated correlations between selected exposure variables. Multivariable regression was then carried out to estimate the adjusted prevalence ratio (PR) as a measure of risk [37, 38]. Three confounders: age, sex and residence, were adjusted for in the multivariable regression model. Since the occurrence of catastrophic costs was high (27%), Poisson’s regression with robust variance was used to [39, 40] give an accurate estimation and interpretation of risk [39–42]. Adjusted prevalence ratios and 95% confidence intervals (*CI*) were derived from the final model. Similarly, an alpha of < 0.05 was considered statistically significant. Post estimation tests were performed to assess the predictive model. Variance inflation factors (VIF) (below 10) was used to assess for multicollinearity. Goodness of fit using the Pearson’s chi-square test with an alpha of < 0.05 was considered significant to reject the model.

A deterministic sensitivity analysis was done using different thresholds (10% and 40%) to measure catastrophic costs, and a conservative estimate of the patients’ direct costs (excluding lost income) [11, 43, 44]. For the alternative scenarios, the analysis estimated the occurrence of catastrophic costs and the significant determinants after regression.

## Results

### Participants

**Fig. 2:**
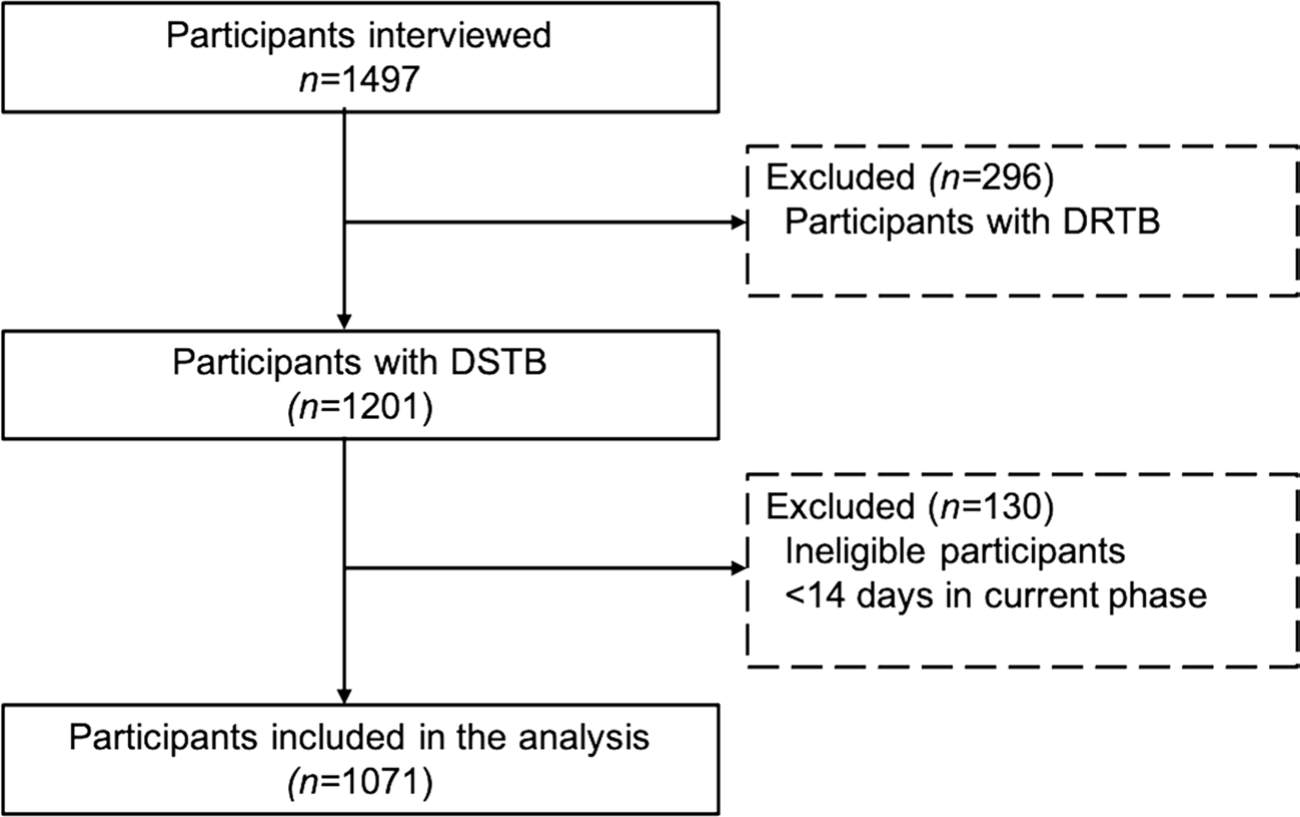
Flow chart detailing participant inclusion. *DSTB* drug-sensitive tuberculosis; *DRTB* drug-resistant tuberculosis

A total of 1071 participants were included in the study after excluding 296 patients with DRTB and 130 patients who were ineligible (Fig. 2).

### Participants baseline characteristics

The mean age of the participants was 31 years, with 15% (*n* = 161) being children under 15 years. Males formed the majority of the respondents (*n* = 665, 62%). One out of ten participants had no formal education (*n* = 127); and a majority had no insurance coverage (*n* = 905, 85%) (Table 2).

**Table 2:** Participant characteristics by occurrence of catastrophic costs at 20% threshold, Kenya 2016 (*n* = 1071)

	Characteristics		Total *n* = 1071	With catastrophic costs* n = 294		without catastrophic costs* n = 777		*P*-value***
				*n*	%**	*n*	%**	
Patient factors	Gender	Female	406	117	39.8	289	37.2	
		Male	665	177	60.2	488	62.8	0.457
	Age	Mean (SD)		31	18.1	31	15.1	0.698^‡^
Household characteristics	Formal education level	Secondary & above	547	155	52.7	392	50.5	
		Primary school & below	397	92	31.3	305	39.3	
		None	127	47	16.0	80	10.3	0.089^†^
	Health insurance	None	905	247	84.0	658	84.7	
		Private	18	6	2.1	12	1.5	
		National Health Insurance	148	41	13.9	107	13.8	0.836^†^
	Residence	Rural	265	71	24.1	194	25.0	
		Urban	806	223	75.9	583	75.0	0.252
	Household size	Median (IQR)		4	(2–6)	4	(2–6)	0.365^†^
	Annual household income (USD)	Median (IQR)		1374	(714–2509)	3081	(1918–5279)	< 0.001^†^
Disease and care-seeking characteristics	TB type	Extra-pulmonary	188	63	21.4	125	16.1	
		Pulmonary	883	231	78.6	651	83.9	0.018
	BMI (*n* = 852)	Mean (SD)		14	8.3	16	7.9	0.023^‡^
	HIV status	Negative	278	81	27.6	197	25.4	
		Positive	769	203	69.4	566	72.8	
		Unknown	24	10	3.4	14	1.8	0.272
	Diabetes status	Yes	20	5	1.7	15	1.9	
		No	1028	283	96.3	745	95.9	
		Unknown	23	6	2.0	17	2.2	0.568
	Treatment category	New case	953	259	88.1	684	88.0	
		Relapse/retreatment	128	35	11.9	93	12.0	0.877
	Facility of treatment	Private	102	35	11.9	67	8.6	
		Public	969	259	88.1	710	91.4	0.102
	Treatment delay > 4 weeks	Yes	180	70	23.8	110	14.2	
		No	891	224	76.2	667	85.8	0.002
	Hospitalization	Yes	6	5	1.7	1	0.1	
		No	1065	289	98.3	776	99.9	0.048
	X-ray done	Yes	589	180	61.2	409	52.6	
		No	482	114	38.8	368	47.4	0.114
	Received nutritional support	Yes	713	192	65.3	521	67.1	
		No	354	100	34.0	254	32.7	
		Unknown	4	2	0.7	2	0.7	0.193

*Where indicated by row title, it represents mean or median, **Where indicated by row title, this represents standard deviation (SD), or interquartile range (IQR), ***Pearson’s Chi Square test for comparison of proportions unless specified

^†^Mann Whitney test

^‡^*t*-test for comparison of means

The socio-demographic characteristics were similar for participants with and without catastrophic costs, except for the expenditure capacity prior to TB illness (Table 2). The participants with catastrophic costs had lower median pre-TB household expenditure USD 1374 (IQR: 714–2509) vs median USD 3081 (IQR: 1918–5279) for those without. There is a significant dose effect relationship between pre-TB household expenditure quintiles and the occurrence of catastrophic costs (lowest quintile: 59.7%, 2nd lowest quintile: 32.9%, middle quintile: 23.6%, 2nd highest quintile: 15.9%, and for the highest wealth quintile: 9.5%).

A comparison of the disease and care-seeking characteristics revealed significant differences for the type of TB, BMI, hospitalization events and the treatment delay (Table 2). The participants with catastrophic costs had a lower nutritional status (mean BMI: 14 vs 16). Further, the subgroup with catastrophic costs had a higher proportion of participants with prolonged treatment delay (26% vs 14%). While only 1% of patients were hospitalized during treatment (*n* = 6), a majority of those hospitalized (*n* = 5, 83%) incurred catastrophic costs.

### Cost consequences and time loss

Participants incurred a median total cost of USD 270 (IQR: 141–502) from the onset of disease till completion of treatment (Table 3). Non-medical expenses accounted for the majority of costs, median USD 173 (IQR: 78–345). A median expenditure of USD 11 (IQR: 0–44) went towards medical costs. Patients spent a median of 29 h for TB care including time spent seeking care in the pre-diagnosis phase (IQR: 17–57). When time was valued, the median indirect costs were USD 36 (IQR: 22–66) (Table 3).

**Table 3:** Costs and time loss by occurrence of catastrophic costs, Kenya 2016

Variable*	All participants ± (*n* = 1071) median (IQR)	With catastrophic costs (*n* = 294) median (IQR)	Without catastrophic costs (*n* = 777) median (IQR)	*P*-value**
Time loss (hours)	29 (17–57)	39 (20–104)	27 (16–47)	< 0.001
Medical costs	11 (0–44)	25 (1.53–100)	9 (0–37)	< 0.001
Non-medical costs	173 (78–345)	306 (159–651)	136 (59–285)	< 0.001
Indirect costs	36 (22–66)	49 (25–139)	33 (21–57)	< 0.001
Total costs	270 (141–502)	567 (299–1149)	212 (120–373)	< 0.001

*All costs in USD, 2020; **Mann–Whitney test

**Fig. 3:**
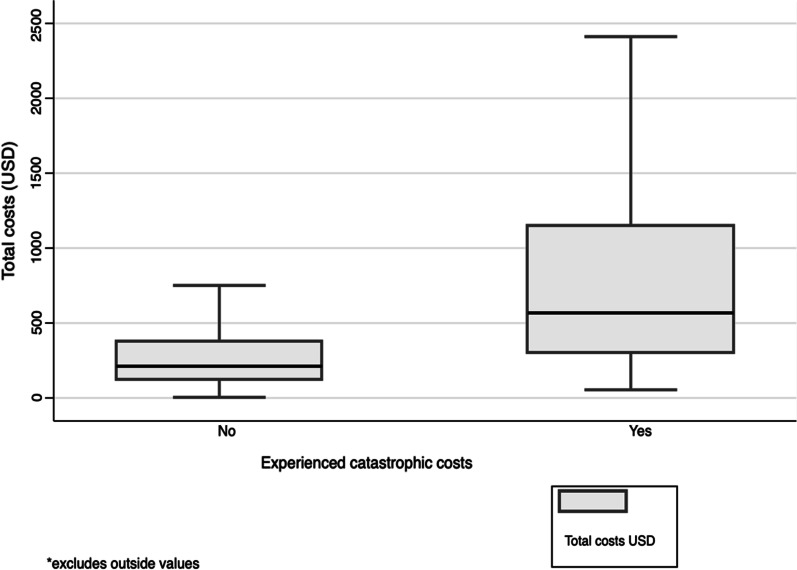
A comparison of total costs by occurrence of catastrophic costs

Participants with catastrophic costs incurred significantly higher costs and time losses (Table 3). The total median costs were USD 567 (IQR: 299–1149) for those with catastrophic costs compared to USD 212 (IQR: 120–373) among those without catastrophic costs. The median costs for the participants with catastrophic costs had a wide distribution (Fig. 3).

Low household pre-TB expenditure, being hospitalized and having a chest X-Ray examination were associated with a higher prevalence of catastrophic costs. Conversely, a better nutritional status and having pulmonary disease (PTB) was associated with a lower risk of catastrophic costs (Table 4).

**Table 4:** Univariable and multivariable Poisson regression of factors associated with catastrophic costs, Kenya patient costs survey (2016)

Variable (*n* = 1071)	Unadjusted prevalence ratio (95% *CI*)	*P*-value	Adjusted prevalence ratio**^‡^ (95% *CI*)	*P*-value***
Hospitalization (ref = not hospitalized)	3.1 (2.1–4.4)	< 0.001	2.8 (1.8–4.5)	< 0.001
Treatment delay (ref = < 4 weeks)	1.6 (1.2–1.9)	< 0.001	1.5 (1.3–1.7)	< 0.001
Type of facility offering treatment (ref = private)	0.8 (0.6–0.9)	0.032	0.8 (0.6–0.9)	0.021
BMI	0.98 (0.97–0.99)	< 0.001	0.97 (0.96–0.98)	< 0.001
Pre-TB HH exp_quintiles (ref = Quintile 5)				
Poorest Q1	6.3 (3.9–10.3)	< 0.001	6.2 (4.0–9.7)	< 0.001
Q2	3.40 (2.1–5.6)	< 0.001	3.4 (2.1–5.3)	< 0.001
Q3	2.5 (1.4– 4.2)	0.002	2.4 (1.5–3.9)	< 0.001
Q4	1.7 (0.9–3.2)	0.111	1.6 (0.9–3.0)	0.110
TB type (ref = EPTB^†^)	0.8 (0.6–1.0)	0.050		
X-ray test (ref = no X-Ray)	1.3 (1.1–1.56)	0.018	1.1 (0.9–1.4)	0.147
Constant			0.19	< 0.001

*CI* confidence interval, *BMI* body mass index, *Pre-TB exp* pre-TB annual HH expenditure quintiles, *Q* quintile, *EPTB* extrapulmonary TB, *Xray* radiological examination

*Table includes only significant variables from univariable analysis, **Adjusted for confounders: age, sex, residence

^‡^Postestimation tests for final model, variance inflation factor (VIF) > 10 for type of facility & TB type. Type of facility maintained in multivariable analysis a priori due to public health significance. Goodness of fit: Pearson’s chi square: non-significant > 0.05

**Fig. 4:**
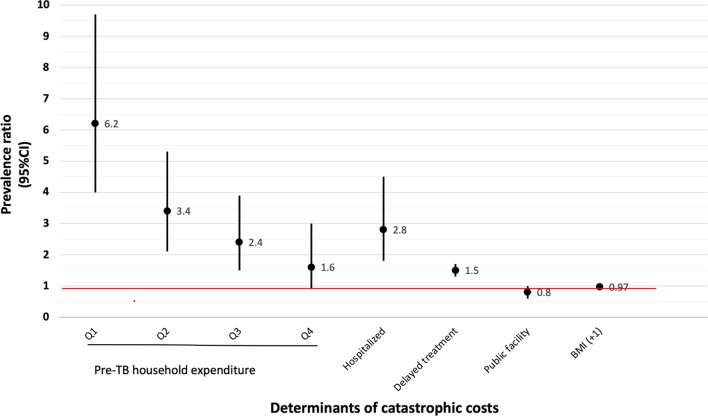
Significant predictors of catastrophic costs

After adjusting for potential confounders and covariates, household expenditure quintiles (pre-TB), hospitalization, treatment delay were associated with a higher incidence of catastrophic costs (Table 4). Lower pre-TB household expenditure was associated with a higher incidence of catastrophic costs. The lowest quintile was 6 times more likely (95% *CI:* 4.0–9.7) to incur catastrophic costs compared to the wealthiest quintile. Being hospitalized during TB treatment had a PR of 2.8 (95% *CI:* 1.8–4.5). Treatment delay of more than four weeks was associated with a PR of 1.5 PR (95% *CI:* 1.3–1.7). Seeking care in a public facility and a higher BMI were protective against incurring catastrophic costs with PR of 0.8 (95% *CI:* 0.6–1.0) and 0.97 (95% *CI:* 0.96–0.98) respectively (Table 4). The graph below depicts the association between covariates and the PR of incurring catastrophic costs (Fig. 4).

There were significant associations between the covariates (Fig. 4). The type of TB was associated with having an X-ray [(Phi coefficient, Phi) = 0.2, *P* < 0.001]. Household expenditure was associated for nutritional status (Cramer’s V = 0.11, *P* = 0.014) and having an X-ray (Cramer’s V = 0.16, *P* < 0.001). Seeking care at a private hospital was associated with hospitalization (Phi = 0.14, *P* < 0.001).

### Sensitivity analysis

Hospitalization, household expenditure and BMI were significant determinants for catastrophic costs in the different scenarios, with changes in the strength of association (PR) (Table 5).

**Table 5:** Deterministic sensitivity analysis with prevalence and determinants of catastrophic costs

Scenario (*n* = 1071)	Occurrence of catastrophic costs		Significant determinants**
	%	*n*	
Base case (BC)*	27	294	Pre TB exp Hospitalization Facility type Treatment delay BMI
10% threshold	54.3	582	Pre TB exp Hospitalization BMI
40% threshold	12.8	137	Similar to base case Age
Conservative estimate, direct costs***	20.9	224	Similar to base case

*Base case represents total costs/household expenditure; threshold of 20%, **Pre-TB exp = pre-TB annual household expenditure quintiles, *BMI* body mass index; ***Represents direct costs/household expenditure; threshold at 20%

At 10% threshold, 54% of the participants incurred catastrophic costs (*n* = 582). At 40% threshold however, 13% (*n* = 137) incurred catastrophic costs and increasing age was an additional predictor. The conservative estimate, using only direct costs had a similar profile for determinants while 21% (*n* = 224) incurred catastrophic costs.

## Discussion

Recognizing the determinants of catastrophic costs could provide an insight into approaches for mitigating catastrophic costs among the vulnerable TB patients and their households. This study confirms that a significant proportion of patients with DSTB (27%) incur catastrophic costs, despite free TB diagnosis and treatment. Further, it demonstrates the association between incurring catastrophic costs and poverty, disease severity and delayed treatment initiation. Low economic status is the most significant factor associated with incurring catastrophic costs. Patients who experience catastrophic costs have a lower economic status (pre-TB) and incur higher costs and productivity losses in relation to economic capacity. Patients with severe disease requiring inpatient care, and those who experience delay before treatment are also at a higher risk of incurring catastrophic costs. On the other hand, seeking care at a public health facility and a better nutritional status are protective factors.

### Catastrophic costs and poverty

Being poor prior to TB illness is associated with the highest incidence of catastrophic costs, resulting in the medical poverty trap. Using household expenditure quintiles in this study demonstrates the higher risk associated with the decreasing economic status. This finding is similar to previous studies in other low- and middle-income settings like Uganda [24], Benin [35] Malawi [45] and China [34]. These findings are also in line with Barasa et al. when considering OOP expenditure for general illness in Kenya [46]. The median age of this study is 31 years representing a young productive age-group. Hence catastrophic costs among the poor can be explained by the loss of earnings during illness and absenteeism, coupled with increased spending on transportation and food as part of the care seeking and treatment process. There is low coverage of social protection measures such as health insurance and insurance against income loss when sick in Kenya [47, 48]. The informal sector, which employed 80% of Kenyans in 2016 [49], is associated with low and irregular pay. The low pay poses a challenge for contributory health insurance schemes [50]. Additionally, paid sick leave is uncommon in the informal sector [51]. The findings of this study indicate that targeting the lower income households for social protection interventions may protect them from catastrophic costs.

### Catastrophic costs and disease severity

Although a small number of participants in this study reported having been hospitalized (*n* = 6), five (83%) of them incurred catastrophic costs. These finding are similar to other studies in Uganda [24], and India [52]. This may be attributed to costs like the bed fee and additional treatment which are not covered by the TB programme. Additionally, family members or treatment supporters may incur OOP costs for transportation, accommodation, in addition to missing out on their income earning activities. That only 0.6% of patients were hospitalized may indicate inaccessibility of services for poor patients. The TB care model in Kenya is predominantly community-based DOTS [22], with hospitalization for those with life-threatening symptoms requiring care such as oxygen therapy or intravenous fluid support. Since there is no literature available on the expected hospitalization rates for TB patients, it is difficult to compare with other settings. Nevertheless, these findings could imply that wealthier patient populations are able to afford inpatient care, compared to the poorer patients. In this study, hospitalization and household expenditure have no significant association. However, there is a significant association between hospitalization and seeking care at a private facility. Previous studies in Kenya also demonstrated pro-rich inequality in access to inpatient care and private facilities utilization for generalized illness [53, 54].

Low BMI is associated with a higher occurrence of catastrophic costs. Similar findings were reported in Ghana [55] and may be explained by the need to spend more on nutritious food and specialized diets [13]. Malnutrition among TB patients is also associated with more severe TB disease [56, 57] and may affect treatment outcomes [58, 59] or even predispose to adverse effects [60], all factors that may increase costs of care. A study in Kenya showed that TB patients who received nutritional support had higher chance of completing their treatment [61].

### Catastrophic costs and health-seeking characteristics

Despite free TB diagnosis and medicines even in private facilities, receiving care in these facilities was associated with a higher incidence of catastrophic costs. Higher costs in private facilities is consistent with findings in Nigeria [62]. This may be explained by extra medical costs at the private facilities which are not included in the NTP package made available to private providers. Continued support for subsidized care at private hospital is important to increase patient access to TB care. However, there is need to explore the extra costs in private facilities and how they hinder access to care.

Delayed treatment initiation is a significant determinant for catastrophic costs in this study. About 24% of participants with catastrophic costs waited longer than four weeks after onset of symptoms to start treatment. Severe symptoms, increased need for hospitalization, more expensive non-TB treatment or even more frequent visits to the facilities may partially explain why delayed treatment initiation was associated [63–65]. Similar findings have been reported in other studies, associated with multiple visits to inappropriate providers; including formal providers that do not have the capacity to screen for TB or are untrained to recognize TB symptoms or informal providers such as traditional healers [24, 35, 66]. A patient pathway analysis conducted in Kenya, showed that although majority of patients (84%) seek care within a formal health facility, only 43% of the facilities had the capacity to diagnose TB and another 45% could support TB treatment [18]. This leads to the health system delay in starting treatment. On the other hand, patient factors such as economic status, beliefs and stigma also contribute to a delay in seeking care [67]. There is a need to further explore contextualized barriers to seeking and receiving care as a preventive measure for catastrophic costs.

## Unexpected findings

This study differed from existing literature regarding the association of catastrophic costs with sex [24, 45], HIV co-infection [24, 52, 62] and household size [21]. In this study, the patient’s sex was not a significant determinant of catastrophic costs. This may be due to the measurement process: Household expenditure and wages to quantify indirect costs were not sensitive to patient’s sex. This observation, may also be attributed to the empowerment of women in Kenya regarding family spending and decision-making [68]. A comparison of gender indices shows significant differences in women decision-making over the household spending and their health [69].

Although HIV is associated with treatment delay and X-ray testing, it was not a significant predictor of catastrophic costs. This is contrary to similar studies conducted in Uganda, Nigeria and India [24, 52, 62]. In Kenya additional social support from the HIV programme is provided [18]. In this study, there is a strong association between HIV coinfection and receiving nutritional support which may have alleviated the food costs and therefore resulting in less costs. These findings may also indicate positive integration of TB/HIV collaborative health service delivery care in Kenya. Finally, household size was not a predictor of catastrophic costs in this study. When DRTB patients were included however, household size was associated with catastrophic costs [21]. Other studies show an association between household size and catastrophic health expenditure for health in Kenya [46]. This may suggest that larger households have a different way of coping with TB costs such as selling assets, borrowing or seeking extra income [54].

## Methodological considerations

This study uses Poisson’s regression methods with robust variance to generate prevalence ratios. This method was chosen to generate an accurate estimation of risk [41, 42] and an accurate interpretation of risk [42]. Logistic regression is associated with an over-estimation of risk for a prevalence greater than 10% [39–41]. Adjusted prevalence ratios and confidence intervals were derived from the final model.

This study took into account both direct and indirect costs, recognizing that time spent seeking care contributes significantly to the burden of TB costs [7]. The indirect costs for this study were calculated using the Human Capital approach due to the low level of formal employment. Although this approach captures all time off work due to the illness and care seeking, it does not take into account household mitigation efforts to compensate for lost income. This may have caused an over-estimation of the productivity losses.

For the denominator, the study used the non-food consumption expenditure as a proxy for household income. This approach provides a better estimate for permanent income in low- and middle-income countries where the rate of formal employment is low [43, 44]. Asset-based wealth was not used since there is a poor correlation with permanent income and tends to overestimates the wealth status of the poor [44, 70].

The study findings can be generalized to DSTB patients in Kenya. Cluster sampling was used in the original survey, taking into account the regional distribution of patients in 2016 and to counter coverage bias. The participants demographic and socio-economic characteristics is representative of the various profiles of DSTB patients in Kenya.

## Study limitations

The study has several limitations. First, the 20% threshold defined by WHO was used to define catastrophic costs. This threshold may underestimate the burden of catastrophic costs in Kenya based on contextual factors such as the level of poverty and ongoing social protection interventions. However, using the 20% threshold ensured comparability with other countries. The study incorporated a sensitivity analysis at 10% and 40% thresholds to check compatibility and ensure reliability of the results. Second, the patient survey enrolment was facility-based, therefore excluding TB patients who did not seek care at facilities or who may have dropped out of the system prior to treatment. This may lead to late presentation and severe disease and at a higher risk of incurring catastrophic costs when they present for TB care. This study did not take these patients and their determinants into account. The Kenya national TB prevalence survey showed that male patients aged 25–34 and 65 years and above delayed seeking care even when symptomatic [17]. Additionally, since costs are a barrier to seeking care, it could have excluded the very poor who do not make it to the health facilities.

This study looked at predictors of OOP costs borne by patients. However, it would be interesting to explore household coping mechanisms such as selling assets and how these affect the identified predictors of catastrophic costs. Documenting intangible costs such as social consequences, stigma and to value quality of life losses is another opportunity for research. A qualitative study at the community-level seeking to explore the impact of the disease among TB affected patients, families and communities would offer a more in-depth perspective into the social and economic consequences of TB disease. A comparison of these study findings with other diseases with a predilection for poverty, such as COVID-19 would inform us about equity and social determinants of health.

## Conclusions

Despite free TB diagnosis and treatment in Kenya, a significant proportion of patients still incur a substantial financial burden. The predictors of catastrophic costs among DSTB patients and their households include pre-TB economic status, treatment delay, disease severity and the type of facility where they received care. There are inequities in experiencing catastrophic costs and the poorest are most likely to suffer the medical poverty trap.

Our study has several implications for policymakers in TB. First, interventions beyond the existing free diagnosis and care are needed to protect the DSTB patients against catastrophic costs. Social protection interventions such as cash transfers, access to health insurance coverage, sickness benefits and vouchers could mitigate against catastrophic costs. NTPs implementing social protection interventions should take these predictors into consideration as a guide to identify the most vulnerable populations. Since patients with severe TB were more likely to experience catastrophic costs, it is crucial to identify them earlier. Strengthening TB case finding is key to protecting DSTB patients against catastrophic costs. Interventions such as increasing the capacity of smaller health facilities to diagnose and treat TB or establishing sputum referral networks and active case finding strategies, could reduce costs due to travel and shorten the delay to treatment. Further, community level interventions including education and engagement about TB and available care options may reduce diagnostic delays.

Whereas an umbrella social protection programme that covers all DSTB patients would be ideal, scarce resources necessitate targeting of those who are most at risk. This study has identified patients who are most vulnerable to catastrophic costs.

## Supplementary Information

**Additional file 1: Additional Table 1.** Summary of Kenya TB burden, 2018.

**Additional file 2: Additional Table 2.** Available data by patient group Kenya Patient cost survey (2016).

## Data Availability

The dataset contains sensitive personal information and is not publicly available. For more information, contact the national TB programme Monitoring and Evaluation unit: aibanr@yahoo.com.

## Abbreviations

BMI: Body mass index
*CI*: Confidence interval
DSTB: Drug-sensitive tuberculosis
DRTB: Drug-resistant tuberculosis
DOTS: Daily Observed Treatment Strategy
EPTB: Extra-pulmonary tuberculosis
FBO: Faith Based Organization
GDP: Gross Domestic Product
HDI: Human development index
IQR: Interquartile range
KDHS: Kenya Demographic and Health Survey
MDR-TB: Multi Drug Resistant tuberculosis
MOH: Ministry of Health
NGO: Non-governmental organization
NTP: National TB Programme
OOP: Out-of-pocket
PCS: Patient Cost Survey
PR: Prevalence ratio
PTB: Pulmonary tuberculosis
Q: Quintile
SD: Standard deviation
TB: Tuberculosis
USD: US Dollar
WHO: World Health Organization
