# Supplementary material for: Determinants of household catastrophic costs for drug sensitive tuberculosis patients in Kenya
Source: Infect Dis Poverty. 2021 Jul 5;10:95. doi: 10.1186/s40249-021-00879-4 (PMC8256229; doi:10.1186/s40249-021-00879-4)
Supplement: Supplementary file 1 — Additional file 1: Additional Table 1. Summary of Kenya TB burden, 2018. [file 40249_2021_879_MOESM1_ESM.pdf]

Additional file

Additional table 1: Summary of Kenya TB burden, 2018*

Indicator	**Number (n)**	**Rate (per 100 000 population)**
TB incidence rate (all forms of TB)	150 000	292
HIV positive TB incidence	40 000	79
Multidrug-/rifampicin-resistant (MDR/RR-TB) incidence	2 300	4.5
HIV negative TB mortality	19 000	38
HIV positive TB mortality	13 000	26
TB cases notified	96 478	64%**

*Source: WHO global report 2019 ** proportion of total incident cases
