# Supplementary material for: Determinants of household catastrophic costs for drug sensitive tuberculosis patients in Kenya
Source: Infect Dis Poverty. 2021 Jul 5;10:95. doi: 10.1186/s40249-021-00879-4 (PMC8256229; doi:10.1186/s40249-021-00879-4)
Supplement: Supplementary file 2 — Additional file 2: Additional Table 2. Available data by patient group Kenya Patient cost survey (2016). [file 40249_2021_879_MOESM2_ESM.pdf]

Additional file 2

Additional table: Available data by patient group Kenya Patient cost survey (2016)

**Data**	**New cases**		**Retreatment Cases**	
	Intensive phase	Continuation phase	Intensive phase	Continuation phase
Personal data / demographic characteristics	√	√	√	√
TB disease information	√	√	√	√
Health Utilization data	√	√	√	√
Time loss and costs before treatment for current diagnosis	√			
Time loss and costs for current TB treatment*	√	√	√	√
Time loss and costs for previous TB treatments			√	√
Time loss& costs for guardian/ treatment supporter	√	√	√	√

√ data collected *The costs were only collected for the current phase of treatment
